# Hyperspectral Imaging as a Tool for Viability Assessment During Normothermic Machine Perfusion of Human Livers: A Proof of Concept Pilot Study

**DOI:** 10.3389/ti.2022.10355

**Published:** 2022-05-16

**Authors:** Margot Fodor, Lukas Lanser, Julia Hofmann, Giorgi Otarashvili, Marlene Pühringer, Benno Cardini, Rupert Oberhuber, Thomas Resch, Annemarie Weissenbacher, Manuel Maglione, Christian Margreiter, Philipp Zelger, Johannes D. Pallua, Dietmar Öfner, Robert Sucher, Theresa Hautz, Stefan Schneeberger

**Affiliations:** ^1^ Department of Visceral, Transplant and Thoracic Surgery, Medical University of Innsbruck, Innsbruck, Austria; ^2^ OrganLife, Organ Regeneration Center of Excellence, Innsbruck, Austria; ^3^ Department of Internal Medicine II, Medical University of Innsbruck, Innsbruck, Austria; ^4^ Department for Hearing, Speech, and Voice Disorders, Medical University of Innsbruck, Innsbruck, Austria; ^5^ University Hospital for Orthopedics and Traumatology, Medical University of Innsbruck, Innsbruck, Austria; ^6^ Department of Visceral, Transplant, Thoracic and Vascular Surgery, Leipzig University Clinic, Leipzig, Germany

**Keywords:** transplantation, perfusion, normothermic, imaging, liver, hyperspectral, machine

## Abstract

Normothermic machine perfusion (NMP) allows for *ex vivo* viability and functional assessment prior to liver transplantation (LT). Hyperspectral imaging represents a suitable, non-invasive method to evaluate tissue morphology and organ perfusion during NMP. Liver allografts were subjected to NMP prior to LT. Serial image acquisition of oxygen saturation levels (StO2), organ hemoglobin (THI), near-infrared perfusion (NIR) and tissue water indices (TWI) through hyperspectral imaging was performed during static cold storage, at 1h, 6h, 12h and at the end of NMP. The readouts were correlated with perfusate parameters at equivalent time points. Twenty-one deceased donor livers were included in the study. Seven (33.0%) were discarded due to poor organ function during NMP. StO2 (*p* < 0.001), THI (*p* < 0.001) and NIR (*p* = 0.002) significantly augmented, from static cold storage (pre-NMP) to NMP end, while TWI dropped (*p* = 0.005) during the observational period. At 12–24h, a significantly higher hemoglobin concentration (THI) in the superficial tissue layers was seen in discarded, compared to transplanted livers (*p* = 0.036). Lactate values at 12h NMP correlated negatively with NIR perfusion index between 12 and 24h NMP and with the delta NIR perfusion index between 1 and 24h (rs = −0.883, *p* = 0.008 for both). Furthermore, NIR and TWI correlated with lactate clearance and pH. This study provides first evidence of feasibility of hyperspectral imaging as a potentially helpful contact-free organ viability assessment tool during liver NMP.

## GRAPHICAL ABSTRACT

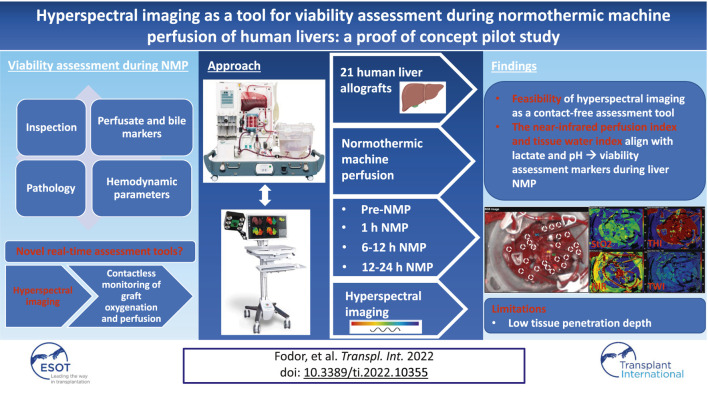

## Introduction

In the light of a shortage of donor liver organs, the use of extended criteria donors (ECD) continues to rise. This poses a risk of increased rates of early allograft dysfunction (EAD), primary non-function (PNF) and biliary complications (1–10). Compared to standard criteria donor grafts, ECD livers are more susceptible towards ischemia-reperfusion injury (IRI). In the light of these developments, machine perfusion (MP) has emerged as a procedure aiming to limit IRI. Normothermic machine perfusion (NMP) is also suitable for prolongation of preservation and a comprehensive assessment of livers *ex-vivo*. While this concept is uniquely appealing, the identification of techniques and biomarkers for a meaningful determination of the quality and function of an organ remains to be established. Essentially, NMP mimics physiologic liver perfusion. During a period of up to 24 h, the liver is accessible for inspection, biopsy, perfusate and bile sampling (11). Contemporarily, viability assessment is performed by measuring biochemical parameters and synthetic function in the perfusate and bile (12–17). Further to this, innovative liver graft viability and injury markers have been applied. However, whether they are acceptable predictors of the outcomes after LT remains to be proven (2, 4, 11, 12). Novel non-invasive methods for the estimation of organ quality during NMP are necessitated. Hyperspectral imaging (HSI) represents a potentially suitable contactless tool to assess tissue morphology and organ perfusion. This technology allows a real-time quantitative evaluation of graft oxygenation and micro-perfusion, as well as organ hemoglobin and water concentration. Previous studies showed that HSI is suitable for monitoring of the oxygen saturation distribution and identifying areas with a reduced oxygen supply (18–20). This may help to detect and quantify impaired, inhomogeneous or deteriorating perfusion (18–27). We herein designed a study demonstrating the feasibility and the potential of HSI in the setting of liver NMP as a non-invasive, simple viability assessment tool.

## Materials and Methods

### Study Design

**FIGURE 1:**
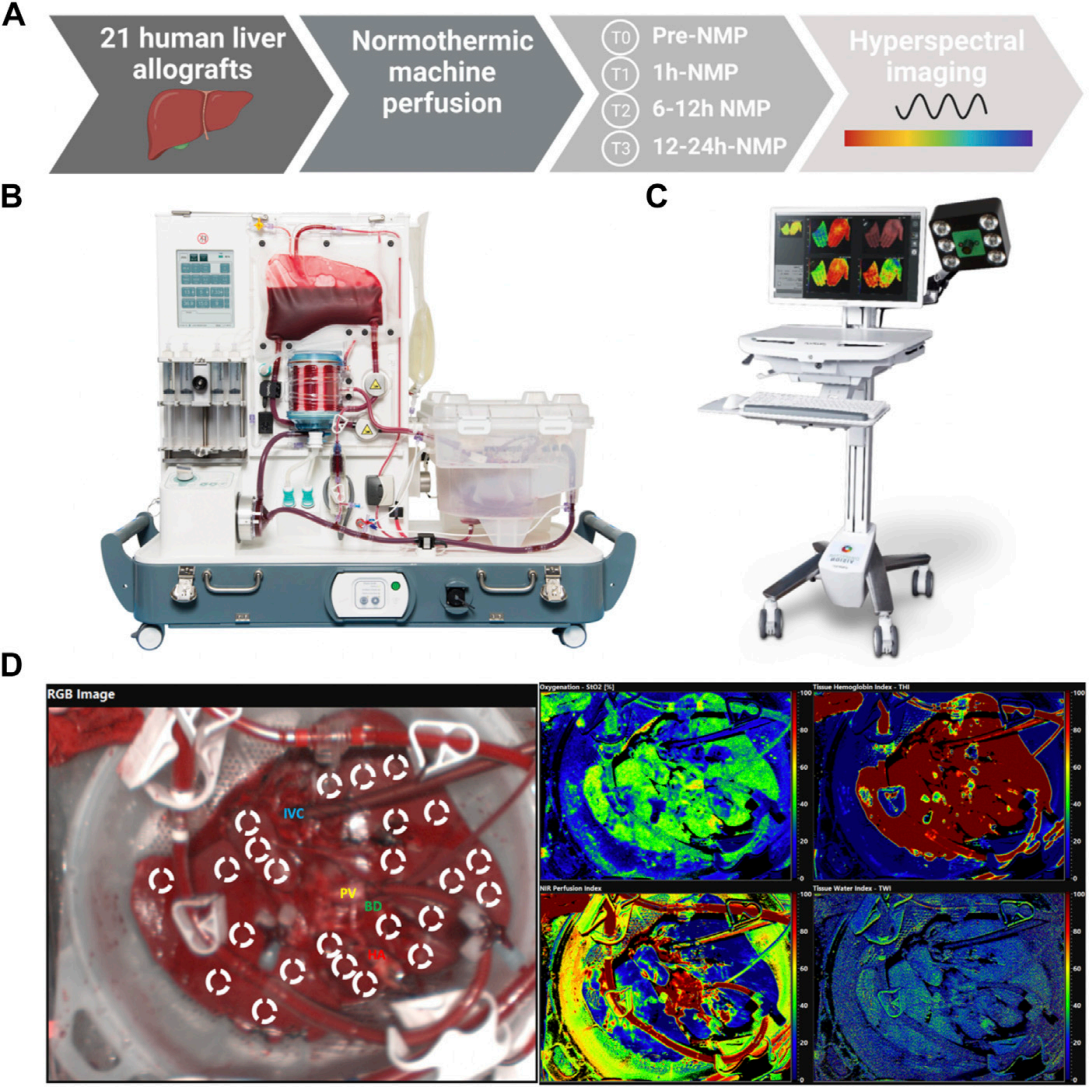
**(A)** Overview describing the methodology and sample collection of the study; **(B)** OrganOx metra® system used for normothermic machine perfusion (14, 16, 52); **(C)** TIVITA® Tissue System used for hyperspectral imaging (21); **(D)** Images acquired during liver normothermic machine perfusion: RGB image, hyperspectral images for oxygenation (StO2), perfusion (NIR perfusion), hemoglobin (THI), and water concentration (TWI), with region of interest (ROI) markers within the parenchyma of liver allografts ICV, Inferior vena cava; PV, Portal vein; HA, Hepatic artery; BD, Bile duct; StO2, Tissue Oxygen Saturation; THI, Tissue Hemoglobin Index; NIR, Near-Infrared Perfusion Index; TWI, Tissue Water Index.

Liver allografts accepted for transplantation were procured and subjected to NMP. The decision to apply NMP at our center was based on a previously developed concept (6). NMP was applied for the following indications: (I) uncertain organ quality (II) complex recipient, and (III) logistics. MP was performed using the OrganOx metra® system according to a local protocol (6), details are specified in the Supplementary File. Perfusion time on the OrganOx metra® system depended on the time required for assessment, decision-making and logistics. The choice to discard or transplant an organ was based on key quality parameters (6, 14): preservation of physiological pH values (7.3–7.45) without sodium bicarbonate supplementation after 2 h of NMP, a prompt decline and maintenance of lactate to physiological values (≤18 mg/dl), as well as bile production and bile pH > 7.45 are considered indicators for appropriate organ function. The decision to transplant or discard a liver graft was made after a minimum of 6 h NMP. Further to this, high aspartate aminotransferase (AST), alanine aminotransferase (ALT) (>20,000), and lactate dehydrogenase (>20,000) levels are calling for caution (1, 6). To assess the dynamics of HSI parameters during liver NMP and their correlation with perfusate parameters, serial measurements were performed before NMP (during static cold storage), at 1, 6, 12 h and at the end of NMP (Figure 1). HSI data points were assessed longitudinally and in reference to the established biomarkers mentioned above. Donor, recipient and NMP characteristics, transplant procedural data as well as post-operative follow-up data were collected.

### Ethics Statement

The study protocol was approved by the local institutional review board.

### Study Population

A total of 21 donor livers were enrolled in this study between December 2020 and May 2021. The majority of these livers were ECD livers. For definition of ECD, the Eurotransplant criteria were applied (28). These include liver grafts with severe macrosteatosis (>30 or >40%), prolonged cold ischemia (>12 h), DCD and high donor age (>80 years). Notably, a number of criteria that could characterize ECDs specifically for LT have been identified, but the impact of each of these remains to be defined (29). From the 21 livers studied in this trial, 14 were transplanted, while seven livers were discarded based on the above-mentioned performance quality criteria during NMP. An overview summarizing the most important characteristics of donors, recipients, liver allografts and MP times are displayed in Table 1.

**TABLE 1:** Demographic data.

	Total (*n* = 21)	Transplanted (*n* = 14)	Not transplanted (n = 7)	*p*-value^a^
**Donor data**				
Age (y)^b^	61 (48–70)	66 (56–70)	46 (43–56)	*p* = 0.031
Gender				*p* = 0.011
• Man	13 (61.9)	6 (42.9)	7 (100)	
• Woman	8 (38.1)	8 (57.1)		
BMI (kg/m^2^)^b^	26 (24–28)	26 (24–28)	28 (23–31)	*p* = 0.585
ICU time (d)^b^	3 (2–7)	4 (2–7)	2 (2–7)	*p* = 0.585
CIT (h)^b^	6 (5–8)	6 (5–8)	7 (5–9)	*p* = 0.856
Cause of death				*p* = 0.290
Cerebrovascular	15 (71.4)	10 (71.4)	5 (71.4)	
Circulatory	2 (9.5)	1 (7.1)	1 (14.3)	
Trauma	1 (4.8)		1 (14.3)	
Other	3 (14.3)	3 (21.4)		
ECD donor	16 (76.2)	10 (71.4)	6 (85.7)	*p* = 0.469
Donor Type				*p* = 1.000
DBD	15 (71.4)	10 (71.4)	5 (71.4)	
DCD	6 (28.6)	4 (28.6)	2 (28.6)	
DRI^b^	2.119 (1.610–2.435)	2.268 (1.728–2.482)	1.760 (1.480–2.220)	*p* = 0.263
Hypertension	7 (33.3)	4 (28.6)	3 (42.9)	*p* = 0.289
Alcohol Abuse	4 (19)	1 (7.1)	3 (42.9)	*p* = 0.102
Malignancy	2 (9.5)	1 (7.1)	1 (14.3)	*p* = 0.599
Steatosis hepatis	11 (52.4)	6 (42.9)	5 (71.4)	*p* = 0.279
• Mild (<40%)	10 (47.6)	5 (35.7)	5 (71.4)	
• Moderate (40%–80%)	1 (4.8)	1 (7.2)	0 (0)	
• Severe (>80%)	0 (0)	0 (0)	0 (0)	
NMP indication				
• Complex recipient	2 (9.5)	2 (14.3)		*p* = 0.293
• Marginal donor	18 (85.7)	11 (78.6)	7 (100)	*p* = 0.186
• Logistics	8 (38.1)	6 (42.9)	2 (28.6)	*p* = 0.525
NMP time (h)^b^	15 (11–20)	15 (13–20)	12 (7–22)	*p* = 0.535
Total preservation time (h)^b^	20 (17–27)	21 (17–27)	19 (9–30)	*p* = 0.799
**Recipient data and post-operative outcome**				
Age (y)^b^		62 (58–65)		
Gender				
• Man		10 (71.4)		
• Woman		4 (28.6)		
BMI (kg/m^2^)^b^		25.7 (21.8–28.2)		
MELD^b^		17 (8–21)		
Time on waiting list (d)^b^		52 (37–197)		
BAR score^b^		7 (7–10)		
BAR score ≥ 8		6 (42.9)		
Total hospital stay (d)^b^		28 (21–46)		
ICU stay (d)^b^		6 (4–18)		
Early allograft dysfunction		6 (42.9)		
MEAF score^b^		5.67 (4.02–6.90)		
L-Graft score^b^		−0.73 (-1.33–0.07)		
Clavien Dindo ≥3		11 (78.6)		
90—days readmission rate (unplanned)		4 (28.6)		
Biliary complications		9 (64.3)		
• ≤ 30 d		6 (42.9)		
• > 30 d		3 (21.4)		
• Biliary leakage		4 (28.6)		
• Anastomotic stricture		4 (28.6)		
• Biliary cast syndrome		1 (7.1)		
Arterial complication		2 (14.3)		
Patient survival (d)^b^		106 (82–163)		
Graft survival (d)^b^		106 (82–163)		
Patient death		2 (14.3)		

Values in parentheses are percentages unless indicated otherwise

a Chi-square for categorical variables and Mann-Whitney-U Test for continuous variables.

b Values are median (i.q.r.).

BMI, body mass index; ICU, intensive care unit; CIT, cold ischemia time; ECD, extended criteria donor; DBD, donation after brain death; DCD, donation after cardiac death; DRI, donor risk index; NMP, normothermic machine perfusion; MELD, Model for End-Stage Liver Disease; BAR, balance of risk; MEAF, model of early allograft function; L-Graft, Liver Graft Assessment Following Transplantation.

### Hyperspectral Imaging of Human Liver Allografts

For the acquisition of HSI data, a contactless and non-ionizing radiation imaging system (TIVITA® Tissue System, Diaspective Vision GmbH, Am Salzhaff, Germany) was used under standardized conditions and previously reported settings (24, 30). The software (TIVITA Suite Tissue) provides a red-green-blue (RGB) image and four false color images illustrating physiologic parameters of the recorded tissue area, which quantified values of the parameters from blue (low values) to red (high values). The relative blood oxygenation in the microcirculation of superficial hepatic tissue layers (approximately 1 mm) is represented by StO2 (%), whereas the near-infrared (NIR) perfusion index (0–100) represents tissue layers in 4–6 mm penetration depth. The indices THI (0–100) and TWI (0–100) display the relative distribution of hemoglobin and water in the investigated tissue area, respectively. Serial HSI measurements were performed according to our center specific NMP protocol: before NMP, at 1h, 6h, 12 h and at the end (with a maximum of 24 h) of NMP. Supplementary Figure S1 show the different perfusion times of the liver grafts. For the assessment protocol, circular areas, representing the ROI (10 mm diameter markers, 3 markers per liver segment), were defined within the acquired hyperspectral images (Figure 1). The index average was calculated from the values collected from the ROI for each image. Details regarding the application of HSI in this analysis are illustrated in the Supplementary File.

### Feasibility and Follow-Up

We primarily assessed the dynamic change of perfusion and oxygenation of liver tissue during NMP. Further to this, we have investigated 1) the differences in HSI dynamics between livers discarded and transplanted as well as 2) the correlations between HSI indices and perfusion parameters.

The clinical follow-up of transplanted patients included the assessment of patient survival, graft survival, Clavien Dindo post-operative complications rate, EAD, Model of Early Allograft Function (MEAF) score, Liver Graft Assessment Following Transplantation (L-Graft) score, biliary and vascular complications, 90 days readmission rate, ICU and total hospital stay.

### Statistical Analysis

To examine the Gaussian distribution, we used the D’Agostino-Pearson normality test. The data were analyzed as proportions and medians with interquartile ranges (IQR), because they were consistent with a skewed distribution. Chi-square and Fisher’s exact tests for categorical variables and Mann-Whitney-U tests for continuous variables were used to compare the HSI values in the transplanted and non-transplanted groups. Ordinal variables were analyzed as continuous variables. Using the t or F distributions, Mann-Whitney-U tests were approximated for ordinal variables. The Friedman test and Sing tests were applied for paired non-parametric tests. To correlate HSI parameters and laboratory values measured in the perfusate during NMP, Spearman rank correlation tests were performed. Two-tailed *p*-values < 0.05 were considered significant throughout the entire analysis. Statistical analysis was performed using SPSS Statistics Version 27.0 for Macintosh (IBM Corporation, Armonk, NY, United States).

## Results

### Patient Characteristics and Flow Parameters During Normothermic Machine Perfusion

During the study period, a total of 21 deceased donor livers were preserved via NMP. Night-time procedures were avoided and NMP time did not exceed 24 h. Seven (33.0%) were discarded after NMP, due to insufficient organ quality and performance. The median donor age was 61 years (48–70 years) and the median Donor Risk Index was 2.119 (1.610–2.435). Cold ischemia time (CIT) was 6 h (5–8 h) and total NMP time was 20 h (17–27 h). Six (28.6%) grafts derived from DCD donors (Maastricht category III), the remaining grafts from DBD donors. Median recipient MELD and Balance of risk (BAR) scores were 17 (8–21) and 7 (7–10), respectively. The median recipient age was 62 years (58–65 years). The median donor age of transplanted vs. discarded livers was 66 (56–70 years) vs. 46 years (43–56 years) (*p* = 0.031). All discarded liver allografts were from male donors, while 8 (57.1%) transplanted liver allografts were from female donors (*p* = 0.011).

The median ICU and total hospital stay were 6 (4–18) and 28 days (21–46), respectively. Six patients (42.9%) developed EAD, the median MEAF and L-Graft scores were 5.67 (4.02–6.90) and −0.73 (−1.33 – (−0.07)). Clavien-Dindo grade ≥3 complications occurred in 11 (78.6%) of 14 patients. Arterial complications occurred in two (14.3%) patients (one anastomotic stricture, one anastomotic aneurysm). Early (≤30 days) biliary complications were detected in six (42.9%) while late biliary complications (>30 days) in three (21.4%) patients. No patients developed non-anastomotic strictures, ischemic type biliary lesions (ITBL) or primary non-function. No patients were listed for re-transplantation. Two patients died due to multi-organ failure. The median follow-up was 106 (82–163) days. Recipient and donor demographics, as well as post-operative outcome parameters are described in Table 1. NMP hepatic artery and portal vein flows were >150 ml/min and >500 ml/min, for all livers during the entire course.

### Perfusion and Oxygenation of the Liver Parenchyma During Normothermic Machine Perfusion

**FIGURE 2:**
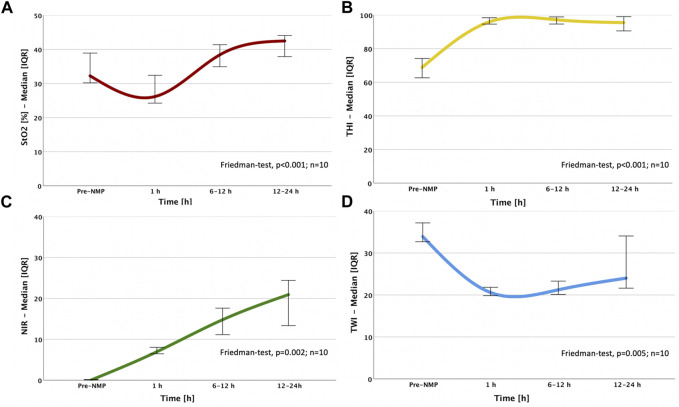
Dynamic changes of HSI indices over NMP time: **(A)** StO2; **(B)** THI; **(C)** NIR; **(D)** TWI; the sample size (*n* = 10) indicates that the Friedman test was calculated based on the ten livers perfused over 12 h and therefore, all NMP time points could be included in the statistical analysis. StO2, Tissue Oxygen Saturation; THI, Tissue Hemoglobin Index; NIR, Near-Infrared Perfusion Index; TWI, Tissue Water Index.

**FIGURE 3:**
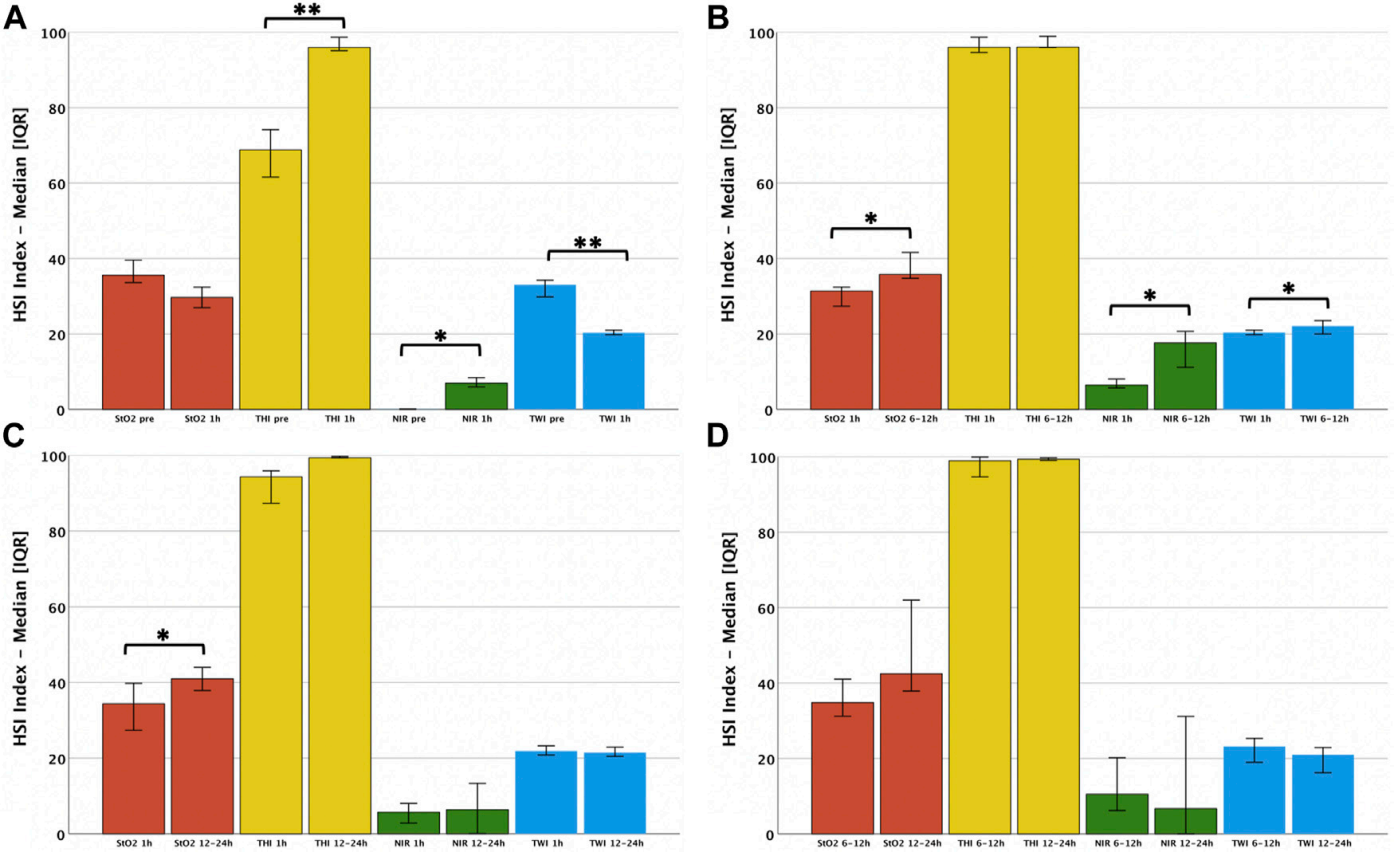
Dynamics of HSI indices between single time points during NMP: **(A)** Pre-NMP to 1 h NMP; **(B)** 1 h NMP to 6–12 h NMP; **(C)** 1 h NMP to 12–24 h NMP; **(D)** 6–12 h NMP to 12–24 h NMP Sing-test: *p < 0.05; **p < 0.01 StO2, Tissue Oxygen Saturation; THI, Tissue Hemoglobin Index; NIR, Near-Infrared Perfusion Index; TWI, Tissue Water Index.

The liver parenchyma was analyzed by HSI in cold-stored organs, at 1, 6, 12 h and at the end of NMP. The StO2, THI and NIR perfusion indices significantly increased (*p* < 0.001, *p* < 0.001 and *p* = 0.002 respectively), while the TWI drastically decreased (*p* = 0.005) during the observational period (Figure 2; Supplementary Table S1). In the interval between static cold storage (pre-NMP) and 1 h NMP, we observed a significant augmentation of the THI and NIR (69 vs. 96, *p* < 0.001 and 0 vs. 7, *p* = 0.003, respectively), while the TWI dropped (33 vs. 20, *p* < 0.001). Contrarily, StO2 mainly remained constant. The dynamics of perfusion and oxygenation over the entire NMP period (between 1 h and 12–24 h) illustrated a significant augmentation of StO2 (31 vs. 39, *p* = 0.006), while the remaining HSI parameters remained stable. A longitudinal assessment of the tissue during NMP showed a substantial increase of the relative blood oxygenation StO2 (31 vs. 41, *p* = 0.008), the NIR perfusion index (7 vs. 17, *p* = 0.008) and the water distribution (TWI) (20 vs. 21, *p* = 0.008) during the first 6 h of NMP, while HSI values remained stable after this time (Figure 3; Supplementary Table S2).

### Discrimination of HSI Dynamics in Transplanted and Discarded Liver Grafts

**FIGURE 4:**
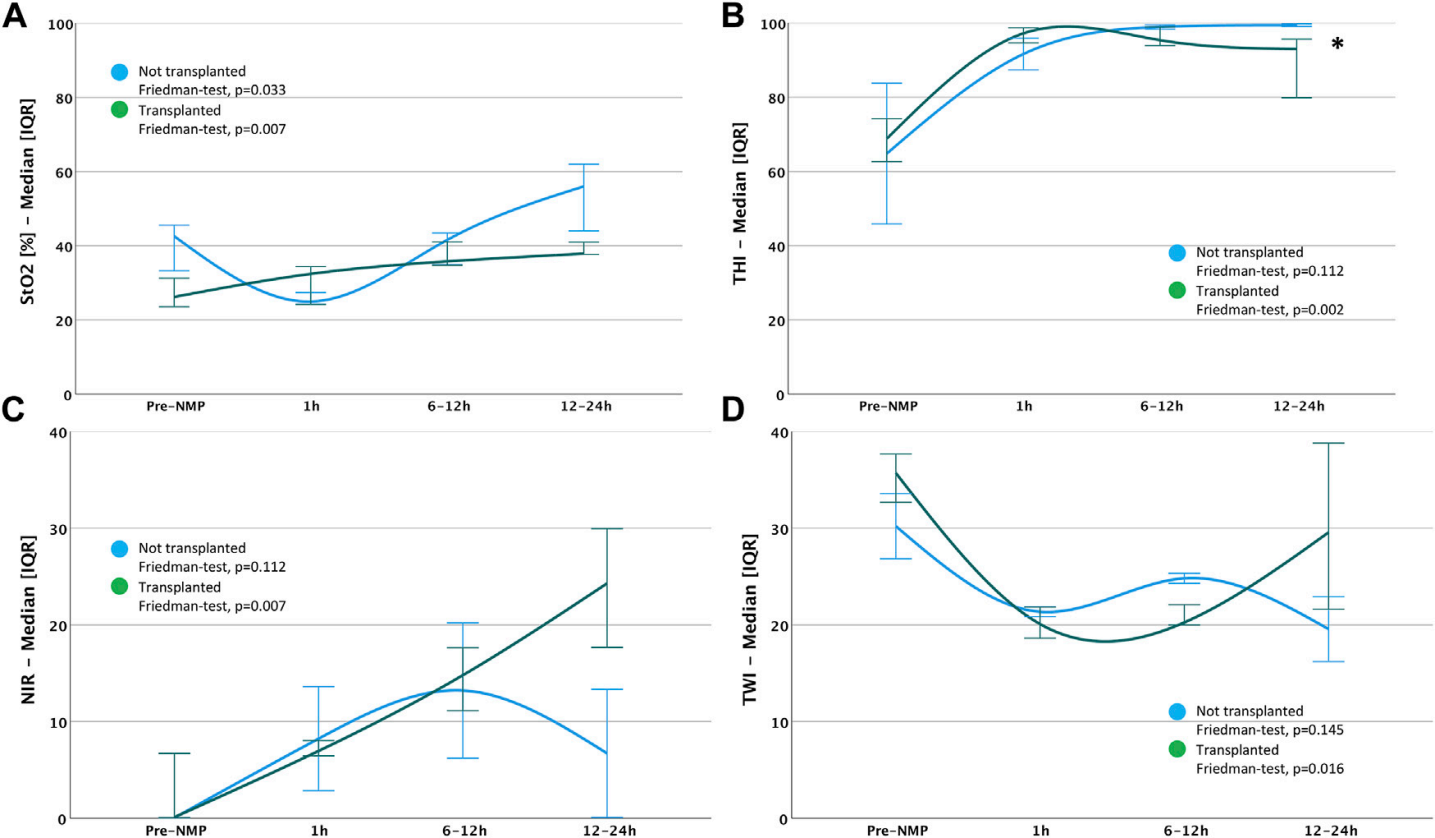
Differences in dynamics of HSI indices between transplanted and not-transplanted liver grafts: **(A)** StO2; **(B)** THI; **(C)** NIR; **(D)** TWI Mann-Whitney-test: *p < 0.05; **p < 0.01 StO2, Tissue Oxygen Saturation; THI, Tissue Hemoglobin Index; NIR, Near-Infrared Perfusion Index; TWI, Tissue Water Index.

Liver allografts subjected to NMP and transplantation revealed a significant escalation of the StO2, THI and NIR perfusion index (*p* = 0.007, *p* = 0.002 and *p* = 0.007, respectively) over the entire observational period, while the tissue water concentration (TWI) drastically decreased (*p* = 0.016). Livers undergoing NMP without subsequent transplantation also displayed a significant augmentation of the relative blood oxygenation (StO2%) (*p* = 0.033). However, the other HSI parameters remained mainly constant during the study period. Notably, at the end of perfusion (12–24 h), a significantly higher hemoglobin concentration (THI) in the superficial tissue layers was seen in discarded, compared to transplanted livers (*p* = 0.036). In contrast, StO2, THI, NIR perfusion index and TWI parameters did not differ during the early course of NMP (Figure 4; Supplementary Tables S3, S4). Discriminations of HSI findings between livers from DBD vs. DCD and SCD vs. ECD, as well as livers with sufficient vs. insufficient lactate clearance during the first 6 hours of NMP were performed. The Supplementary File displays these additional findings (Supplementary Figures S2–S4; Supplementary Tables S5–S10).

### Correlation of HSI Indices With Perfusion Parameters During Normothermic Machine Perfusion

**FIGURE 5:**
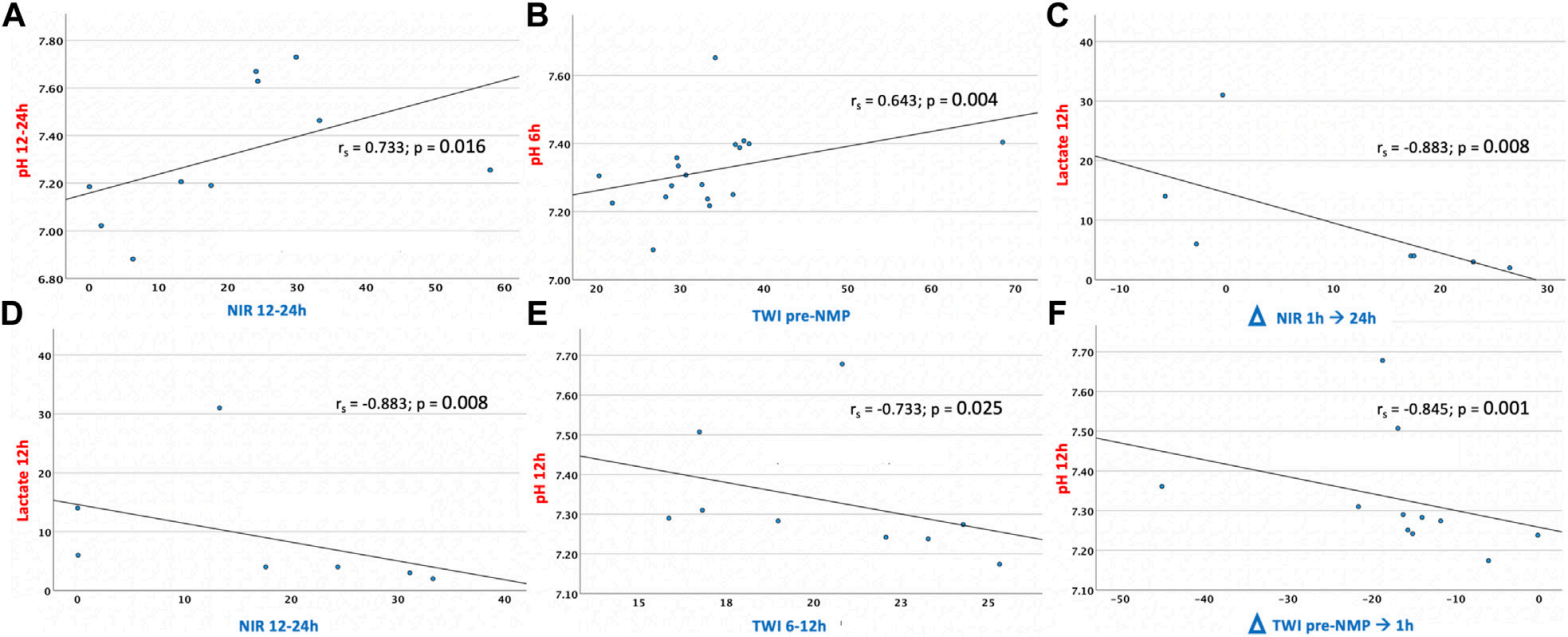
Significant correlations of HSI indices and clinical applied perfusion parameters*:* **(A)** NIR 12–24 h NMP/pH 12–24 h NMP; **(B)** TWI pre-NMP/pH 6 h NMP; **(C)** Delta NIR 1–24 h NMP/Lactate 12 h NMP; **(D)** NIR 12–24 h NMP/Lactate 12 h NMP; **(E)** TWI 6–12 h NMP/pH 12 h NMP; **(F)** Delta TWI pre-NMP-1h NMP/pH 12 h NMP Spearman´s correlation: r_s_, Spearman’s Rank Correlation Coefficient; *p < 0.05; **p < 0.01 NIR, Near-Infrared Perfusion Index; TWI, Tissue Water Index.

There is currently limited evidence about the predictive value of individual perfusion parameters (12). Several biomarkers have been proposed to determine optimal clinical and metabolic liver responses during *ex vivo* NMP, including perfusate lactate clearance, or maintenance of a stable perfusate pH value (31). Lactate has traditionally been used as a marker of sepsis. Lactatemia can subsequently develop in tissue hypoxia (31). In this context, the liver is responsible for removing about 50% of circulating serum lactate, which rises in the liver in case of reduced blood flow/oxygen delivery. In line with the consideration that lactate should be interpreted as a surrogate marker of hypoxic injury and impaired hepatocyte functionality (32–34), our data displayed a negative correlation of increasing lactate values at 12 h NMP with a high NIR perfusion index between 12 and 24 h NMP and with an improved delta NIR perfusion index between 1 and 24 h (rs = −0.883, *p* = 0.008 for both). The perfusate pH has been introduced as a viability criterium in the context of NMP, given the association with lactic acidosis, most commonly resulting from an imbalance between oxygen delivery and oxygen demand (12). Concomitant to this assumption, our analysis revealed a positive correlation of perfusate pH with the NIR perfusion index over 12 h NMP (rs = 0.733, *p* = 0.016). The TWI concomitated a decrease in lactatemia and the rising pH. Liver oedema and the related parenchymal damage as detected with HSI during cold storage decreased during NMP. In accordance, the TWI between 6 and 12 h and between cold storage and 1 h NMP were negatively associated with the pH at 12 h (rs = −0.733, *p* = 0.025 and rs = −0.845, *p* = 0.001, respectively), while a high TWI during static cold storage correlated with a high pH at 6 h (rs = 0.643, *p* = 0.004), (Figure 5; Supplementary Tables S11–S13). These findings suggest that the NIR perfusion index and the TWI are potential markers to estimate the severity of impaired perfusion and oxygenation in livers during NMP.

## Discussion

This pilot study was conducted with the intent to investigate HSI in the clinical setting of liver NMP. NMP allows to push the boundaries of organ transplantation, including the use of ECD grafts and longer preservation times (12, 13, 15, 35–37). HSI represents a user friendly imaging technology allowing for a quick and contactless, real-time viability assessment (22). *In vivo*, HSI can detect alterations at the early stages of NMP. While intra-operative haemodynamic monitoring has been limited to systemic measurements, a more organ-specific approach reflecting local oxygen delivery and microcirculatory perfusion has gained interest (38–40). In the field of hepatobiliary surgery, different imaging techniques were tested in order to evaluate liver parenchymal perfusion (41, 42). Indocyanine green fluorescence was examined as a technology aiding with intraoperative navigation, useful to detect patients at risk for developing EAD after LT (42). Moreover, this method may be utilized as tool to define boundaries of ischaemic areas by capillary flow diffusion in gastrointestinal surgery (38). Intraoperative changes in the oxygenation state of liver grafts were previously measured by near infrared spectroscopy. Mean hepatic oxygen saturation of hemoglobin in the liver was positively correlated with portal flow rate, indicating heterogeneous tissue oxygenation. This parameter was also predictive of EAD (43). Sidestream dark field imaging, a microscopic technique using polarized light to visualize erythrocytes through capillaries, was experimented as non-invasive method to visualize the microvessel architecture (38). In contrast to commonly used methods for determining the oxygenation status, HSI allows a pixel-wise analysis of chemical changes. The additional information on oxygenation status and perfusion quality, might facilitate the decision-making process in transplantation (18–20, 22, 23, 30). Currently utilized HSI parameters like StO2, NIR perfusion index and THI might be of lesser importance if measured during cold storage. However, in the context of MP, HSI may provide useful data on organ viability and performance (22). Moreover, the continuous monitoring of liver micro-perfusion, oxygenation and water content offers an early identification of functional/technical limitations during MP. For the entire observational period, we observed a significant increase in oxygen saturation, tissue hemoglobin concentration and micro-perfusion, while the organ water amount drastically diminished. Furthermore, a subgroup discrimination between transplanted and discarded liver allografts showed an enhanced micro-perfusion in transplanted grafts, mainly after 6–12 h NMP. We observed that tissue oxygenation and micro-perfusion are specifically augmented during the first 12 h of NMP, while lesser dynamic changes were displayed in the late phase of NMP. Current liver graft evaluation is either based on scoring systems involving donor and recipient parameters, or on the invasive assessment of the parenchyma (44–51). Histopathologic examination of liver biopsies represents the current gold standard in the evaluation of liver quality in transplantation, however, several limitations such as time requirement, work-up procedure, reproducibility, intraoperative variance, inappropriate sampling, as well as the invasive nature of the retrieval represent important limitations. Further, histopathology may not always be a reliable indicator of graft quality, since this procedure only captures a snapshot of the morphological but not the functional condition (5). Other assessment technologies include perfusate/bile flow biomarkers as well as hydrodynamic parameters (11, 12). It remains to be determined, if they can be used as long-term indicators of graft outcomes (11). For livers rejected for transplantation based on particular viability criteria, no postoperative data are available and the direct comparison remains elusive (12). A decision-making process based on NMP endpoints poses the risk of incorrectly discarding organs suitable for transplantation. A definitive viability validation would require a well-powered multicenter randomized controlled trial (11). In an attempt to assess if HSI indices correspond with the perfusate biomarkers, our primary findings suggest, that NIR and TWI align with lactate and pH, considered as viability assessment markers during NMP. Based on the limited number of cases analyzed in this study, no conclusions toward an immediate clinical application can be drawn. HSI cannot replace histopathology or the viability markers currently applied. While clinical endpoints in LT trials such as EAD, MEAF and L-Graft score were applied in this study, the restricted number of transplanted patients and the selection applied through assessment during NMP did not permit the identification of discrimination towards the outcome by HSI.

The strengths of the HSI technology as applied during NMP are the immediate applicability and the comprehensive assessment of the perfusion state of an organ over the entire exposed surface (22). Integrating of a real-time imaging procedure into a clinical MP setting would require optimal acquisition distance settings and automated use under sterile conditions (22). Further to the use during NMP, utilization during donor surgery for quality assessment before cold perfusion and procurement could be of interest (30).

In addition to the small cohort analyzed in this study, the different perfusion times and the overall heterogeneity of the liver grafts represent apparent limitations. Further to these, HSI has a relatively low tissue penetration depth, which precludes the detection of injuries in deeper regions, or the potential transcutaneous measurement after transplantation. All in all, HSI during NMP appears promising and feasible and its apparent simplicity makes it attractive for clinical use, but validation in large clinical trials is needed before establishing routine application. All analyses are explorative and *p*-values ≤ 0.05 were termed significant for descriptive reasons only.

To the best of our knowledge, HSI has not yet been applied previously in the field of liver NMP. We herein proved the technical feasibility of the combination of HSI and NMP. This real-time perfusion imaging may contribute to pre-transplant viability assessment.

## Data Availability

The original contributions presented in the study are included in the article/Supplementary Material, further inquiries can be directed to the corresponding author.

## Abbreviations

ALT, alanine aminotransferase; AST, aspartate aminotransferase; BAR, balance of risk score; CIT, cold ischemia time; DBD, donation after brain death; DCD, donation after cardiac death; EAD, early graft dysfunction; ECD, extended criteria donors; HSI, hyperspectral imaging; HTK, histidine-tryptophan-ketoglutarate; ICU, intensive care unit; IGL-1, Institut Georges Lopez; IQR, interquartile range; IRI, ischemia reperfusion injury; ITBL, ischemic type biliary lesions; L-GrAFT, Liver Graft Assessment Following Transplantation; LT, Liver transplantation; MEAF, Model for Early Allograft Function; MELD, model for end-stage liver disease; MP, machine perfusion; NIR, near-infrared perfusion index; NMP, normothermic machine perfusion; PNF, primary non-function; RGB, red-green-blue; ROI, region of interest; StO2 (%), relative blood oxygenation index; THI, tissue hemoglobin index; TWI, tissue water index; UW, University of Wisconsin.
