# Supplementary material for: Hyperspectral Imaging as a Tool for Viability Assessment During Normothermic Machine Perfusion of Human Livers: A Proof of Concept Pilot Study
Source: Transpl Int. 2022 May 16;35:10355. doi: 10.3389/ti.2022.10355 (PMC9150258; doi:10.3389/ti.2022.10355)
Supplement: Supplementary file 1 [file DataSheet1.docx]

**Supplementary File**

**Methods**

*Normothermic machine perfusion*

Livers were previously flushed in situ with University of Wisconsin (UW) (n=2), Institute Georges Lopez (IGL-1) (n=2), or Histidine-tryptophan-ketoglutarate (HTK) (n=17) solution and initially preserved for transportation in the respective preservation fluid on ice. Upon arrival, livers were prepared back-table for NMP, flushed with 500 mL Gelofusine® (B. Braun, Germany) for cannula drenching and then connected to the perfusion device. The perfusate consisted of three units of type O leukocyte-depleted packed red blood cells mixed with 500 mL Gelofusine® (B. Braun, Germany). The infusions as per the manufacturers protocol include (i) bile salt (sodium taurocholate, New Zealand Pharmaceuticals, New Zealand), (ii) insulin (Actrapid®, Novo Nordisk, UK), (iii) heparin (CP Pharmaceuticals, UK), and (iv) prostacyclin (Flolan®, Glaxo, UK). An hourly assessed glucose level regulates a variable-rate of fat-free total parenteral nutrition (Nutriflex, B Braun, Sheffield, UK). A standardized perfusion and assessment protocol was designed, including perfusate blood gas and serial biochemical analyses of perfusate samples for liver function parameters [1].

*Indications for NMP*

According to a previously developed center specific protocol [1], NMP was applied for the following indications:

(I) Donor-related indications: NMP may be clinically relevant in extended criteria organs (donor age > 65 years, elevated risk for transmission of a disease, eg, hepatitis b/c; donor body mass index [BMI] > 30 kg/m2; total bilirubin > 3 mg/dL; serum sodium > 165 mmol/L; hospitalization in intensive care unit [ICU] > 7 d; hepatic steatosis > 40%, acute hemodynamic deterioration with risk of organ loss including donation after circulatory determination of death (DCD) livers. Especially in combination with expected prolonged ischemia times, NMP allows better graft preservation and quality assessment.

(II) Recipient-related indications: In cases of surgically highly complex recipients or high-risk patients, NMP is valuable in order to relieve time pressure and reallocate the organ in case hepatectomy is considered impossible.

(III) Logistic-related indications: An extended preservation time has a significant implication on the logistics of the surgical procedure. This allows to better prepare the recipient with a complex medical condition for the surgical procedure. Additionally, NMP reduces the need for parallel surgeries and is helpful in the light of human resource management and working hour restrictions [1].

*Hyperspectral imaging of human liver allografts*

For the acquisition of HSI data, a contactless and non-ionizing radiation imaging system (TIVITA® Tissue System, Diaspective Vision GmbH, Am Salzhaff, Germany) was used under standardized conditions and previously reported settings [2, 3]. After removing the lid of the box containing the liver, the HSI camera was positioned exactly 50 cm above the region of interest (ROI). To avoid artefacts during the HSI acquisition, all ambient light was switched off for a brief period of the measurement. The illumination of the liver was performed by six integrated halogen spots, enabling spectral data acquisition in the visible and near-infrared spectral range from 500 to 1000 nm. Effective pixels were 640 x 480 (x-, y-axis). For exact positioning, an included electro-optical distance measurement system was used. Specific wavelength spikes corresponded to biological and molecular conditions at defined tissue spots and depths, and information on the respective liver tissue could be achieved. After a computation time of fewer than ten seconds, the software (TIVITA Suite Tissue) provides a red-green-blue (RGB) image and four false color images illustrating physiologic parameters of the recorded tissue area, which quantified values of the parameters from blue (low values) to red (high values). The relative blood oxygenation in the microcirculation of superficial hepatic tissue layers (approximately 1 mm) is represented by StO2 (%), whereas the near-infrared (NIR) perfusion index (0 – 100) represents tissue layers in 4 – 6 mm penetration depth. The indices THI (0 – 100) and TWI (0 – 100) display the relative distribution of hemoglobin and water in the investigated tissue area, respectively. Besides these indices, distinctive reflectance spectra can be plotted for a ROI. A detailed description of the system can be found in the study by Holmer et al [4].

Given its contactless and rapid applicability, HSI measurements did not interfere with the NMP procedure (about 10 seconds for recording and the near-“real-time” option of visualization and interpretation). Serial HSI measurements were performed according to our center specific NMP protocol: before NMP, at 1h, 6h, 12h and at the end (with a maximum of 24h) of NMP. All measurements were performed by the surgical staff based on a center-specific protocol, including a theoretical introduction, followed by two hands-on training sessions of fifteen minutes, respectively.

Acquired RGB and color images were collected during NMP and stored for further analysis. For the assessment protocol, circular areas, representing the ROI (10 mm diameter markers, 3 markers per liver segment), were defined within the acquired hyperspectral images (figure 1). The index average was calculated from the values collected from the ROI for each image.

**Figure S1:** Perfusion duration of the 21 livers included in the study cohort (minimal NMP time: 6h; maximal NMP time: 24h)


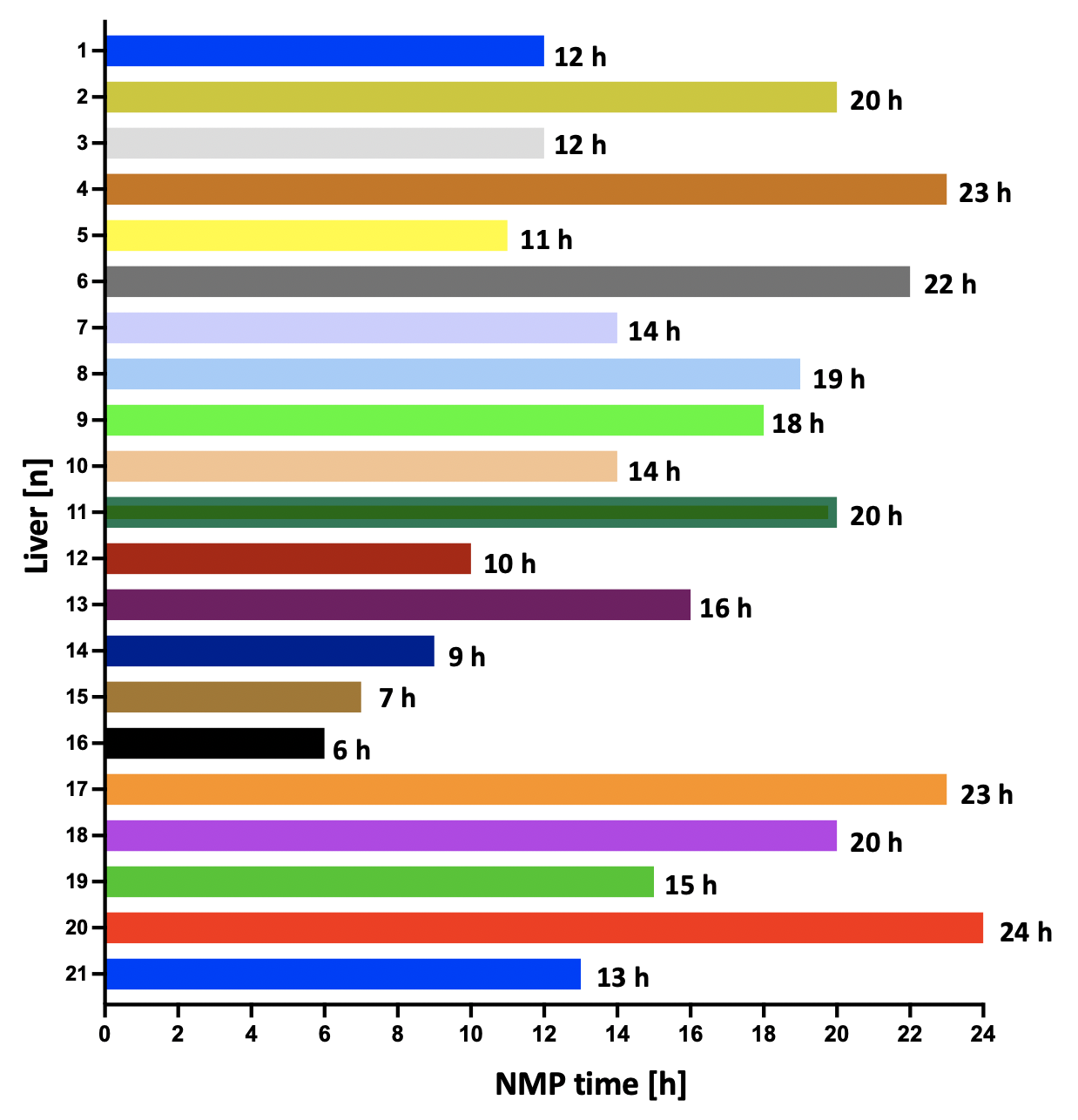


*NMP, Normothermic Machine Perfusion*

**Figure S2**: Differences in dynamics of HSI indices between donation after brain death and donation after cardiac death liver grafts: (A) StO2; (B) THI; (C) NIR; (D) TWI

*Mann-Whitney-test: * p<0.05; ** p<0.01*

*StO2, Tissue Oxygen Saturation; THI, Tissue Hemoglobin Index; NIR, Near-Infrared Perfusion Index; TWI, Tissue Water Index*

**Figure S3**: Differences in dynamics of HSI indices between standard criteria donor and extended criteria donor liver grafts: (A) StO2; (B) THI; (C) NIR; (D) TWI

*Mann-Whitney-test: * p<0.05; ** p<0.01*

*StO2, Tissue Oxygen Saturation; THI, Tissue Hemoglobin Index; NIR, Near-Infrared Perfusion Index; TWI, Tissue Water Index*

**Figure S4**: Differences in dynamics of HSI indices between liver grafts displaying a rapid (≤ 2.5 mmol/l in the first 6h) or slow (> 2.5 mmol/l in the first 6h)/rising lactate level

*Mann-Whitney-test: * p<0.05; ** p<0.01*

*StO2, Tissue Oxygen Saturation; THI, Tissue Hemoglobin Index; NIR, Near-Infrared Perfusion Index; TWI, Tissue Water Index*

**Table S1:** Dynamic changes of HSI indices over NMP time: significance levels (p-value: Friedman-Test)

|  | p-value (Friedman-test) |
| --- | --- |
| **StO2%** | **< 0.001** |
| **THI** | **< 0.001** |
| **NIR** | **0.002** |
| **TWI** | **0.005** |

*HSI, Hyperspectral Imaging; NMP, Normothermic Machine Perfusion; StO2, Tissue Oxygen Saturation; THI, Tissue Hemoglobin Index; NIR, Near-Infrared Perfusion Index; TWI, Tissue Water Index*

**Table S2:** Dynamics of HSI indices between single time points during NMP: significance levels (p-value: Sign-Test)

|  | **Pre-NMP -> 1h** | **1h -> 12-24h** | **1h -> 6-12h** | **6-12h -> 12-24h** |
| --- | --- | --- | --- | --- |
| **StO2%** | 0.263 | **0.006** | **0.008** | 0.344 |
| **THI** | **< 0.001** | 1.000 | 1.000 | 0.344 |
| **NIR** | **0.003** | 0.388 | **0.008** | 1.000 |
| **TWI** | **< 0.001** | 0.388 | **0.008** | 0.754 |

*HSI, Hyperspectral Imaging; NMP, Normothermic Machine Perfusion; StO2, Tissue Oxygen Saturation; THI, Tissue Hemoglobin Index; NIR, Near-Infrared Perfusion Index; TWI, Tissue Water Index*

**Table S3:** Dynamic changes of HSI indices over NMP time: courses of transplanted and not-transplanted liver grafts: significance levels (p-value: Friedman-test)

|  | **Transplanted livers (n=14)** | **Non-transplanted livers (n=7)** |
| --- | --- | --- |
| **StO2%** | **0.007** | **0.033** |
| **THI** | **0.002** | 0.112 |
| **NIR** | **0.007** | 0.112 |
| **TWI** | **0.016** | 0.145 |

*HSI, Hyperspectral Imaging; NMP, Normothermic Machine Perfusion; StO2, Tissue Oxygen Saturation; THI, Tissue Hemoglobin Index; NIR, Near-Infrared Perfusion Index; TWI, Tissue Water Index*

**Table S4:** HSI index values over NMP time: differences between transplanted and not-transplanted liver grafts (p-value: Mann-Whitney-U Test)

|  | **Total**  **(n=21)** | **Transplanted**  **(n=14)** | **Not transplanted**  **(n=7)** | **p-value**  **#** |
| --- | --- | --- | --- | --- |
| **StO2 [%]** | | | | |
| StO2 pre-NMP | 36 (31 - 43) | 35 (30 - 40) | 39 (33 - 46) | p = 0.444 |
| StO2 1h | 31 (25 - 37) | 31 (25 - 35) | 27 (24 - 55) | p = 0.856 |
| StO2 6h-12h | 41 (34 - 43) | 38 (32 - 43) | 43 (35 - 46) | p = 0.213 |
| StO2 12h-24h | 39 (34 - 50) | 38 (34 - 41) | 50 (34 - 62) | p = 0.310 |
| **THI** | | | | |
| THI pre-NMP | 69 (52 - 77) | 72 (62 - 79) | 48 (36 - 74) | p = 0.109 |
| THI 1h | 96 (93 - 99) | 97 (95 - 100) | 93 (86 - 99) | p = 0.197 |
| THI 6h-12h | 97 (95 - 99) | 97 (94 - 99) | 97 (96 - 99) | p = 1.000 |
| THI 12h-24h | 95 (80 - 99) | 91 (80 - 96) | 100 (99 - 100) | **p = 0.036** |
| **NIR** | | | | |
| NIR pre-NMP | 0 (0 - 4) | 0 (0 - 7) | 0 (0 - 2) | p = 0.904 |
| NIR 1h | 7 (4 - 14) | 7 (5 - 18) | 6 (1 - 14) | p = 0.743 |
| NIR 6h-12h | 17 (10 - 22) | 17 (10 - 28) | 20 (3 - 21) | p = 0.659 |
| NIR 12h-24h | 21 (4 - 31) | 24 (18 - 31) | 6 (0 - 13) | p = 0.145 |
| **TWI** | | | | |
| TWI pre-NMP | 33 (29 - 37) | 35 (31 - 38) | 29 (28 - 30) | p = 0.062 |
| TWI 1h | 20 (19 - 22) | 20 (17 - 22) | 21 (20 - 24) | p = 0.322 |
| TWI 6h-12h | 21 (19 - 24) | 20 (19 - 22) | 24 (24 - 25) | p = 0.083 |
| TWI 12h-24h | 26 (22 - 40) | 34 (22 - 40) | 23 (16 - 26) | p = 0.282 |

*Values in parentheses are medians (i.q.r.) unless indicated otherwise; # Mann-Whitney-U Test*

*HSI, Hyperspectral Imaging; NMP, Normothermic Machine Perfusion; StO2, Tissue Oxygen Saturation; THI, Tissue Hemoglobin Index; NIR, Near-Infrared Perfusion Index; TWI, Tissue Water Index*

**Table S5:** Dynamic changes of HSI indices over NMP time: courses of donation after brain death and donation after cardiac death liver grafts: significance levels (p-value: Friedman-test)

|  | **DBD (n=15)** | **DCD (n=6)** |
| --- | --- | --- |
| **StO2%** | **0.035** | **0.019** |
| **THI** | **0.010** | 0.122 |
| **NIR** | **0.034** | 0.072 |
| **TWI** | **0.006** | 0.532 |

*HSI, Hyperspectral Imaging; NMP, Normothermic Machine Perfusion; StO2, Tissue Oxygen Saturation; THI, Tissue Hemoglobin Index; NIR, Near-Infrared Perfusion Index; TWI, Tissue Water Index*

**Table S6:** HSI index values over NMP time: differences between donation after brain death and donation after cardiac death liver grafts (p-value: Mann-Whitney-U Test)

|  | **Total**  **(n=21)** | **DBD**  **(n=15)** | **DCD**  **(n=6)** | **p-value**  **#** |
| --- | --- | --- | --- | --- |
| **StO2 [%]** | | | | |
| StO2 pre-NMP | 36 (31 - 43) | 37 (33 - 44) | 33 (26 - 43) | p = 0.444 |
| StO2 1h | 31 (25 - 37) | 31 (25 - 40) | 28 (24 - 34) | p = 0.569 |
| StO2 6h-12h | 41 (34 - 43) | 42 (35 - 43) | 35 (31 - 41) | p = 0.335 |
| StO2 12h-24h | 39 (34 - 50) | 38 (33 - 59) | 41 (38 - 44) | p = 0.808 |
| **THI** | | | | |
| THI pre-NMP | 69 (52 - 77) | 69 (55 - 74) | 65 (51 - 83) | p = 0.779 |
| THI 1h | 96 (93 - 99) | 95 (90 - 99) | 98 (96 - 100) | p = 0.178 |
| THI 6h-12h | 97 (95 - 99) | 97 (94 - 99) | 97 (96 - 100) | p = 0.750 |
| THI 12h-24h | 95 (80 - 99) | 95 (80 - 99) | 99 (64 - 100) | p = 0.727 |
| **NIR** | | | | |
| NIR pre-NMP | 0 (0 - 4) | 0 (0 - 7) | 0 (0 - 2) | p = 0.547 |
| NIR 1h | 7 (4 - 14) | 7 (1 - 14) | 6 (4 - 19) | p = 0.791 |
| NIR 6h-12h | 17 (10 - 22) | 18 (11 - 21) | 10 (6 – 32) | p = 0.703 |
| NIR 12h-24h | 21 (4 - 31) | 18 (6 - 30) | 24 (0 - 31) | p = 1.000 |
| **TWI** | | | | |
| TWI pre-NMP | 33 (29 - 37) | 34 (30 - 37) | 29 (27 - 33) | p = 0.207 |
| TWI 1h | 20 (19 - 22) | 20 (19 - 22) | 21 (17 - 23) | p = 0.910 |
| TWI 6h-12h | 21 (19 - 24) | 20 (19 - 24) | 23 (21- 25) | p = 0.437 |
| TWI 12h-24h | 26 (22 - 40) | 26 (23 - 39) | 20 (16 - 47) | p = 0.482 |

*Values in parentheses are medians (i.q.r.) unless indicated otherwise; # Mann-Whitney-U Test*

*StO2, Tissue Oxygen Saturation; THI, Tissue Hemoglobin Index; NIR, Near-Infrared Perfusion Index; TWI, Tissue Water Index*

**Table S7:** Dynamic changes of HSI indices over NMP time: courses of standard criteria and extended criteria donor liver grafts: significance levels (p-value: Friedman-test)

|  | **SCD (n=5)** | **ECD (n=16)** |
| --- | --- | --- |
| **StO2%** | 0.145 | **0.005** |
| **THI** | 0.241 | **0.005** |
| **NIR** | 0.241 | **< 0.001** |
| **TWI** | 0.145 | **0.027** |

*HSI, Hyperspectral Imaging; NMP, Normothermic Machine Perfusion; StO2, Tissue Oxygen Saturation; THI, Tissue Hemoglobin Index; NIR, Near-Infrared Perfusion Index; TWI, Tissue Water Index*

**Table S8:** HSI index values over NMP time: differences between standard criteria and extended criteria donor liver grafts (p-value: Mann-Whitney-U Test)

|  | **Total**  **(n=21)** | **SCD**  **(n=5)** | **ECD**  **(n=16)** | **p-value**  **#** |
| --- | --- | --- | --- | --- |
| **StO2 [%]** | | | | |
| StO2 pre-NMP | 36 (31 - 43) | 34 (33 - 34) | 39 (30 - 46) | p = 0.497 |
| StO2 1h | 31 (25 - 37) | 31 (27 - 31) | 30 (25 - 44) | p = 0.603 |
| StO2 6h-12h | 41 (34 - 43) | 42 (41 - 43) | 36 (34 - 43) | p = 0.566 |
| StO2 12h-24h | 39 (34 - 50) | 50 (44 - 56) | 38 (34 - 44) | p = 0.273 |
| **THI** | | | | |
| THI pre-NMP | 69 (52 - 77) | 74 (55 - 74) | 67 (51 - 83) | p = 0.866 |
| THI 1h | 96 (93 - 99) | 95 (90 - 99) | 96 (94 - 99) | p = 0.548 |
| THI 6h-12h | 97 (95 - 99) | 99 (96 - 99) | 96 (95 - 100) | p = 0.849 |
| THI 12h-24h | 95 (80 - 99) | 88 (80 - 96) | 97 (80 - 99) | p = 0.606 |
| **NIR** | | | | |
| NIR pre-NMP | 0 (0 - 4) | 0 (0 - 0) | 0 (0 - 7) | p = 0.800 |
| NIR 1h | 7 (4 - 14) | 4 (1 - 7) | 8 (5 - 16) | p = 0.275 |
| NIR 6h-12h | 17 (10 - 22) | 10 (2 - 22) | 18 (10 - 28) | p = 0.498 |
| NIR 12h-24h | 21 (4 - 31) | 26 (18 - 33) | 19 (2 - 30) | p = 0.485 |
| **TWI** | | | | |
| TWI pre-NMP | 33 (29 - 37) | 31 (29 - 36) | 33 (28 - 38) | p = 0.866 |
| TWI 1h | 20 (19 - 22) | 16 (13 - 20) | 21 (19 - 23) | **p = 0.011** |
| TWI 6h-12h | 21 (19 - 24) | 20 (17 - 21) | 23 (20 - 24) | p = 0.095 |
| TWI 12h-24h | 26 (22 - 40) | 33 (25 - 40) | 25 (21 - 39) | p = 0.606 |

*Values in parentheses are medians (i.q.r.) unless indicated otherwise; # Mann-Whitney-U Test*

*StO2, Tissue Oxygen Saturation; THI, Tissue Hemoglobin Index; NIR, Near-Infrared Perfusion Index; TWI, Tissue Water Index*

**Table S9:** Dynamic changes of HSI indices over NMP time: courses of liver grafts displaying a rapid (≤ 2.5 mmol/l in the first 6h) or slow (> 2.5 mmol/l in the first 6h)/rising lactate level: significance levels (p-value: Friedman-test)

|  | **> 2.5 mmol/l (n=11)** | **≤ 2.5 mmol/l (n=10)** |
| --- | --- | --- |
| **StO2%** | **< 0.001** | **n.a.** |
| **THI** | **0.007** | **0.013** |
| **NIR** | 0.212 | **0.012** |
| **TWI** | **0.035** | **0.044** |

*HSI, Hyperspectral Imaging; NMP, Normothermic Machine Perfusion; StO2, Tissue Oxygen Saturation; THI, Tissue Hemoglobin Index; NIR, Near-Infrared Perfusion Index; TWI, Tissue Water Index*

**Table S10:** HSI index values over NMP time: differences between liver grafts displaying a rapid (≤ 2.5 mmol/l in the first 6h) or slow (> 2.5 mmol/l in the first 6h)/rising lactate level (p-value: Mann-Whitney-U Test)

|  | **Total**  **(n=21)** | **≤ 2.5 mmol/l (n=11)** | **> 2.5 mmol/l (n=10)** | **p-value**  **#** |
| --- | --- | --- | --- | --- |
| **StO2 [%]** | | | | |
| StO2 pre-NMP | 36 (31 - 43) | 39 (35 - 44) | 33 (26 - 43) | p = 0.175 |
| StO2 1h | 31 (25 - 37) | 38 (28 - 52) | 27 (24 - 32) | **p = 0.036** |
| StO2 6h-12h | 41 (34 - 43) | 41 (29 - 49) | 41 (35 - 43) | p = 0.930 |
| StO2 12h-24h | 39 (34 - 50) | 34 (28 - 41) | 44 (38 - 56) | p = 0.145 |
| **THI** | | | | |
| THI pre-NMP | 69 (52 - 77) | 63 (54 - 70) | 74 (46 - 83) | p = 0.412 |
| THI 1h | 96 (93 - 99) | 97 (94 - 99) | 96 (90 - 100) | p = 0.973 |
| THI 6h-12h | 97 (95 - 99) | 95 (92 - 96) | 99 (96 - 100) | **p = 0.044** |
| THI 12h-24h | 95 (80 - 99) | 85 (72 - 97) | 99 (97 - 100) | p = 0.154 |
| **NIR** | | | | |
| NIR pre-NMP | 0 (0 - 4) | 0 (0 - 7) | 0 (0 - 0) | p = 0.370 |
| NIR 1h | 7 (4 - 14) | 8 (6 - 19) | 6 (3 - 8) | p = 0.282 |
| NIR 6h-12h | 17 (10 - 22) | 18 (13 - 28) | 10 (6 - 21) | p = 0.258 |
| NIR 12h-24h | 21 (4 - 31) | 21 (4 - 27) | 22 (7 - 32) | p = 0.808 |
| **TWI** | | | | |
| TWI pre-NMP | 33 (29 - 37) | 37 (33 - 38) | 30 (28 - 34) | p = 0.112 |
| TWI 1h | 20 (19 - 22) | 21 (19 - 24) | 20 (17 - 21) | p = 0.114 |
| TWI 6h-12h | 21 (19 - 24) | 20 (19 - 26) | 22 (19 - 24) | p = 0.791 |
| TWI 12h-24h | 26 (22 - 40) | 36 (24 - 41) | 22 (18 - 24) | **p = 0.048** |

*Values in parentheses are medians (i.q.r.) unless indicated otherwise; # Mann-Whitney-U Test*

*StO2, Tissue Oxygen Saturation; THI, Tissue Hemoglobin Index; NIR, Near-Infrared Perfusion Index; TWI, Tissue Water Index*

**Table S11:** Correlation analysis displaying significant associations between HSI indices and clinical used viability criteria during NMP (p-value: Spearman correlation analysis)

|  | **Spearman correlation** | |
| --- | --- | --- |
|  | Spearman-rank  coefficient | p-value |
| **NIR 12-24h - pH 12-24h** | 0.733 | 0.016 |
| **NIR 12-24h - lactate 12h** | -0.883 | 0.008 |
| **TWI pre-NMP - pH 6h** | 0.643 | 0.004 |
| **TWI 6-12h - pH 12h** | -0.733 | 0.025 |
| **Delta NIR 1-24h - lactate 12h** | -0.883 | 0.008 |
| **Delta TWI pre NMP-1h - pH 12h** | -0.845 | 0.001 |

*HSI, Hyperspectral Imaging; NMP, Normothermic Machine Perfusion; NIR, Near-Infrared Perfusion Index; TWI, Tissue Water Index*

**Table S12:** Overview of the correlation analysis displaying associations between HSI indices and clinical used viability criteria during NMP (p-value: Spearman correlation analysis)

|  | **StO2**  **pre NMP** | **StO2**  **1h** | **StO2**  **6-12** | **StO2**  **12-24** | **THI**  **pre NMP** | **THI**  **1h** | **THI**  **6-12** | **THI**  **12-24** | **NIR**  **pre NMP** | **NIR**  **1h** | **NIR**  **6-12** | **NIR**  **12-24** | **TWI**  **pre NMP** | **TWI**  **1h** | **TWI**  **6-12** | **TWI**  **12-24** |
| --- | --- | --- | --- | --- | --- | --- | --- | --- | --- | --- | --- | --- | --- | --- | --- | --- |
| **ALT**  **1h** | 0.186 | 0.060 | -0.040 | 0.014 | -0.313 | -0.041 | **-.486*** | -0.028 | 0.379 | -0.011 | 0.038 | 0.007 | -0.301 | 0.331 | 0.290 | 0.238 |
| **mg/dl** | 0.431 | 0.797 | 0.874 | 0.966 | 0.179 | 0.860 | **0.041** | 0.931 | 0.100 | 0.962 | 0.880 | 0.983 | 0.198 | 0.143 | 0.243 | 0.457 |
| **ALT**  **6h** | 0.170 | -0.235 | -0.240 | 0.291 | -0.232 | -0.001 | -0.434 | -0.064 | 0.270 | -0.102 | 0.074 | -0.182 | -0.335 | 0.095 | 0.201 | 0.064 |
| **mg/dl** | 0.486 | 0.333 | 0.353 | 0.385 | 0.340 | 0.997 | 0.082 | 0.853 | 0.264 | 0.678 | 0.787 | 0.593 | 0.161 | 0.700 | 0.439 | 0.853 |
| **ALT 12h** | -0.105 | 0.168 | 0.079 | 0.667 | 0.042 | -0.403 | -0.418 | -0.548 | 0.248 | -0.014 | 0.236 | 0.095 | -0.420 | -0.049 | 0.103 | 0.405 |
| **mg/dl** | 0.746 | 0.602 | 0.829 | 0.071 | 0.897 | 0.194 | 0.229 | 0.160 | 0.437 | 0.966 | 0.484 | 0.823 | 0.175 | 0.880 | 0.777 | 0.320 |
| **ALT end** | 0.020 | 0.091 | -0.348 | -0.218 | -0.007 | -0.232 | -0.319 | 0.236 | 0.389 | -0.116 | 0.094 | -0.136 | -0.181 | 0.333 | 0.152 | 0.000 |
| **mg/dl** | 0.938 | 0.710 | 0.171 | 0.519 | 0.977 | 0.339 | 0.213 | 0.484 | 0.111 | 0.637 | 0.729 | 0.689 | 0.473 | 0.163 | 0.560 | 1.000 |
| **AST**  **1h** | 0.162 | 0.017 | -0.018 | 0.035 | -0.263 | -0.084 | -0.453 | 0.140 | 0.328 | -0.027 | 0.042 | -0.147 | -0.311 | 0.304 | 0.271 | 0.105 |
| **mg/dl** | 0.494 | 0.942 | 0.945 | 0.914 | 0.262 | 0.718 | 0.059 | 0.665 | 0.158 | 0.909 | 0.868 | 0.649 | 0.182 | 0.180 | 0.276 | 0.746 |
| **AST**  **6h** | 0.204 | -0.267 | -0.213 | 0.291 | -0.221 | 0.014 | -0.446 | -0.091 | 0.289 | -0.025 | 0.103 | -0.136 | -0.321 | 0.121 | 0.252 | 0.073 |
| **mg/dl** | 0.403 | 0.270 | 0.411 | 0.385 | 0.363 | 0.954 | 0.073 | 0.790 | 0.230 | 0.918 | 0.704 | 0.689 | 0.180 | 0.622 | 0.328 | 0.832 |
| **AST**  **12h** | -0.063 | 0.189 | 0.103 | 0.690 | 0.007 | -0.431 | -0.503 | -0.524 | 0.227 | -0.035 | 0.255 | -0.048 | -0.441 | -0.084 | 0.067 | 0.429 |
| **mg/dl** | 0.846 | 0.557 | 0.777 | 0.058 | 0.983 | 0.162 | 0.138 | 0.183 | 0.478 | 0.914 | 0.450 | 0.911 | 0.152 | 0.795 | 0.855 | 0.289 |
| **AST**  **end** | 0.096 | 0.007 | -0.309 | -0.191 | -0.059 | -0.137 | -0.419 | 0.218 | 0.437 | 0.027 | 0.162 | -0.091 | -0.234 | 0.400 | 0.265 | 0.073 |
| **mg/dl** | 0.705 | 0.977 | 0.228 | 0.574 | 0.817 | 0.576 | 0.094 | 0.519 | 0.069 | 0.912 | 0.549 | 0.790 | 0.349 | 0.090 | 0.305 | 0.832 |
| **LDH**  **1h** | 0.194 | -0.022 | -0.106 | -0.084 | -0.266 | -0.060 | -0.381 | 0.070 | 0.334 | -0.030 | -0.094 | -0.049 | -0.239 | 0.297 | 0.319 | 0.063 |
| **mg/dl** | 0.413 | 0.924 | 0.675 | 0.795 | 0.257 | 0.795 | 0.119 | 0.829 | 0.150 | 0.898 | 0.711 | 0.880 | 0.310 | 0.190 | 0.197 | 0.846 |
| **LDH**  **6h** | 0.246 | -0.261 | -0.272 | 0.109 | -0.247 | 0.079 | -0.407 | -0.145 | 0.321 | -0.013 | 0.091 | -0.082 | -0.282 | 0.132 | 0.287 | 0.055 |
| **mg/dl** | 0.311 | 0.280 | 0.291 | 0.750 | 0.307 | 0.748 | 0.105 | 0.670 | 0.180 | 0.957 | 0.737 | 0.811 | 0.241 | 0.591 | 0.264 | 0.873 |
| **LDH**  **12h** | -0.049 | 0.028 | -0.176 | 0.571 | -0.014 | -0.298 | -0.285 | -0.333 | 0.227 | -0.042 | 0.127 | -0.167 | -0.462 | 0.021 | 0.273 | 0.167 |
| **mg/dl** | 0.880 | 0.931 | 0.627 | 0.139 | 0.966 | 0.347 | 0.425 | 0.420 | 0.478 | 0.897 | 0.709 | 0.693 | 0.131 | 0.948 | 0.446 | 0.693 |
| **LDH**  **end** | 0.065 | -0.011 | -0.385 | -0.309 | -0.028 | -0.059 | -0.324 | 0.127 | 0.458 | -0.096 | 0.071 | 0.045 | -0.193 | 0.312 | 0.208 | 0.082 |
| **mg/dl** | 0.798 | 0.966 | 0.127 | 0.355 | 0.913 | 0.811 | 0.205 | 0.709 | 0.056 | 0.697 | 0.795 | 0.894 | 0.443 | 0.193 | 0.422 | 0.811 |
| **ph**  **1h** | -0.497 | -0.126 | -0.266 | 0.214 | 0.399 | 0.399 | 0.434 | -0.452 | 0.022 | 0.495 | -0.112 | 0.643 | 0.483 | 0.011 | 0.063 | -0.190 |
| **mg/dl** | 0.101 | 0.681 | 0.404 | 0.610 | 0.199 | 0.177 | 0.159 | 0.260 | 0.947 | 0.086 | 0.729 | 0.086 | 0.112 | 0.972 | 0.846 | 0.651 |
| **ph**  **6h** | 0.061 | 0.379 | 0.179 | -0.364 | -0.030 | 0.135 | 0.094 | -0.030 | -0.148 | -0.051 | 0.111 | -0.115 | **.643**** | 0.088 | -0.291 | 0.115 |
| **mg/dl** | 0.810 | 0.121 | 0.506 | 0.272 | 0.906 | 0.593 | 0.729 | 0.934 | 0.558 | 0.842 | 0.694 | 0.751 | **0.004** | 0.729 | 0.274 | 0.751 |
| **ph**  **12h** | -0.400 | -0.182 | 0.150 | 0.190 | 0.118 | 0.269 | -0.250 | -0.321 | -0.417 | -0.409 | -0.297 | 0.143 | 0.255 | -0.482 | **-.733*** | 0.429 |
| **mg/dl** | 0.223 | 0.593 | 0.700 | 0.651 | 0.729 | 0.424 | 0.516 | 0.482 | 0.202 | 0.212 | 0.405 | 0.760 | 0.450 | 0.133 | **0.025** | 0.337 |
| **ph**  **end** | -0.368 | -0.074 | 0.115 | 0.167 | -0.082 | 0.399 | -0.154 | -0.467 | 0.251 | 0.305 | 0.421 | **.733*** | -0.300 | 0.179 | 0.220 | 0.200 |
| **mg/dl** | 0.177 | 0.787 | 0.707 | 0.693 | 0.771 | 0.126 | 0.616 | 0.174 | 0.367 | 0.251 | 0.118 | **0.016** | 0.277 | 0.506 | 0.471 | 0.580 |
| **Lactate 1h** | 0.153 | 0.120 | 0.200 | 0.063 | -0.360 | -0.026 | -0.039 | **.620*** | -0.163 | -0.130 | 0.052 | **-.687*** | -0.110 | 0.353 | 0.413 | -0.354 |
| **mg/dl** | 0.520 | 0.604 | 0.425 | 0.846 | 0.119 | 0.912 | 0.877 | **0.032** | 0.493 | 0.574 | 0.839 | **0.014** | 0.645 | 0.117 | 0.088 | 0.259 |
| **Lactate 6h** | -0.153 | -0.150 | 0.041 | 0.045 | -0.108 | 0.080 | 0.437 | **.669*** | 0.044 | -0.258 | 0.093 | -0.313 | -0.201 | 0.151 | 0.249 | **-.632*** |
| **mg/dl** | 0.544 | 0.552 | 0.879 | 0.894 | 0.671 | 0.753 | 0.091 | **0.034** | 0.862 | 0.302 | 0.741 | 0.379 | 0.424 | 0.550 | 0.351 | **0.050** |
| **Lactate 12h** | 0.050 | 0.041 | 0.301 | 0.635 | -0.123 | -0.437 | -0.201 | 0.378 | -0.205 | 0.041 | 0.256 | **-.883**** | -0.087 | -0.064 | 0.059 | -0.234 |
| **mg/dl** | 0.883 | 0.905 | 0.431 | 0.091 | 0.718 | 0.179 | 0.604 | 0.403 | 0.546 | 0.905 | 0.475 | **0.008** | 0.800 | 0.852 | 0.881 | 0.613 |
| **Lactate end** | 0.077 | 0.356 | 0.460 | -0.098 | -0.300 | -0.129 | -0.086 | 0.246 | 0.203 | -0.215 | 0.141 | -0.327 | -0.287 | 0.069 | 0.064 | 0.021 |
| **mg/dl** | 0.748 | 0.113 | 0.055 | 0.762 | 0.198 | 0.576 | 0.735 | 0.441 | 0.390 | 0.350 | 0.578 | 0.300 | 0.220 | 0.767 | 0.801 | 0.948 |

*Spearman-rank correlation coefficient with according p-value. Statistically, significant correlations are marked in bold*

*ALT,* Alanine Aminotransferase*; AST, Aspartate Aminotransferase; LDH, lactate dehydrogenase; StO2, Tissue Oxygen Saturation; THI, Tissue Hemoglobin Index; NIR, Near-Infrared Perfusion Index; TWI, Tissue Water Index*

**Table S13:** Overview of the correlation analysis displaying associations between delta HSI indices and clinical used viability criteria during NMP (p-value: Spearman correlation analysis)

|  | **StO2 pre NMP-1h** | **THI**  **pre NMP-1h** | **NIR**  **pre NMP-1h** | **TWI**  **pre NMP-1h** | **StO2**  **pre NMP-6h/12h** | **THI**  **pre NMP-6h/12h** | **NIR**  **pre NMP-6h/12h** | **TWI**  **pre NMP-6h/12h** | **StO2**  **1-6h/12h** | **THI**  **1-6h/12h** | **NIR**  **1-6h/12h** | **TWI**  **1-6h/12h** | **StO2**  **1-12h/24h** | **THI**  **1-12h/24h** | **NIR**  **1-12h/24h** | **TWI**  **1-12h/24h** |
| --- | --- | --- | --- | --- | --- | --- | --- | --- | --- | --- | --- | --- | --- | --- | --- | --- |
| **ALT**  **1h** | -0.197 | 0.308 | 0.006 | 0.350 | -0.176 | 0.224 | 0.318 | 0.285 | 0.006 | 0.309 | 0.227 | -0.042 | 0.357 | -0.095 | 0.167 | 0.429 |
| **mg/dl** | 0.405 | 0.186 | 0.980 | 0.130 | 0.627 | 0.533 | 0.340 | 0.425 | 0.987 | 0.385 | 0.502 | 0.907 | 0.385 | 0.823 | 0.693 | 0.289 |
| **ALT**  **6h** | -0.321 | 0.235 | -0.140 | 0.377 | -0.248 | 0.273 | 0.327 | 0.297 | 0.018 | 0.285 | 0.255 | -0.055 | 0.310 | -0.048 | 0.024 | 0.476 |
| **mg/dl** | 0.180 | 0.333 | 0.566 | 0.111 | 0.489 | 0.446 | 0.326 | 0.405 | 0.960 | 0.425 | 0.450 | 0.881 | 0.456 | 0.911 | 0.955 | 0.233 |
| **ALT**  **12h** | 0.147 | -0.042 | 0.063 | 0.517 | -0.245 | 0.039 | 0.164 | 0.081 | 0.005 | -0.034 | 0.047 | -0.211 | -0.309 | 0.327 | 0.082 | 0.045 |
| **mg/dl** | 0.649 | 0.897 | 0.846 | 0.085 | 0.343 | 0.881 | 0.558 | 0.758 | 0.985 | 0.896 | 0.863 | 0.417 | 0.355 | 0.326 | 0.811 | 0.894 |
| **ALT end** | -0.092 | 0.013 | -0.017 | 0.358 | -0.387 | 0.096 | 0.211 | 0.157 | 0.152 | -0.181 | -0.024 | -0.208 | -0.236 | 0.345 | -0.036 | -0.064 |
| **mg/dl** | 0.717 | 0.958 | 0.948 | 0.145 | 0.125 | 0.715 | 0.451 | 0.548 | 0.560 | 0.486 | 0.931 | 0.422 | 0.484 | 0.298 | 0.915 | 0.853 |
| **AST**  **1h** | -0.223 | 0.254 | 0.010 | 0.356 | -0.248 | 0.224 | 0.300 | 0.345 | -0.079 | 0.236 | 0.145 | 0.236 | 0.452 | -0.214 | -0.095 | 0.167 |
| **mg/dl** | 0.346 | 0.280 | 0.967 | 0.123 | 0.489 | 0.533 | 0.370 | 0.328 | 0.829 | 0.511 | 0.670 | 0.511 | 0.260 | 0.610 | 0.823 | 0.693 |
| **AST**  **6h** | -0.370 | 0.223 | -0.075 | 0.391 | -0.414 | 0.042 | 0.079 | 0.074 | 0.042 | -0.228 | 0.012 | -0.152 | -0.227 | 0.200 | 0.182 | -0.064 |
| **mg/dl** | 0.119 | 0.359 | 0.762 | 0.098 | 0.098 | 0.874 | 0.781 | 0.779 | 0.874 | 0.379 | 0.966 | 0.560 | 0.502 | 0.555 | 0.593 | 0.853 |
| **AST**  **12h** | 0.133 | -0.014 | 0.056 | 0.524 | 0.367 | -0.333 | -0.358 | -0.583 | 0.233 | -0.400 | -0.079 | 0.050 | 0.286 | -0.071 | 0.250 | 0.429 |
| **mg/dl** | 0.681 | 0.966 | 0.863 | 0.080 | 0.332 | 0.381 | 0.310 | 0.099 | 0.546 | 0.286 | 0.829 | 0.898 | 0.493 | 0.879 | 0.589 | 0.337 |
| **AST**  **end** | -0.238 | 0.059 | 0.116 | 0.432 | 0.121 | 0.044 | 0.275 | 0.330 | 0.253 | -0.275 | -0.021 | -0.511 | 0.167 | -0.394 | 0.394 | 0.164 |
| **mg/dl** | 0.341 | 0.817 | 0.648 | 0.073 | 0.694 | 0.887 | 0.342 | 0.271 | 0.405 | 0.364 | 0.940 | 0.074 | 0.693 | 0.260 | 0.260 | 0.651 |
| **LDH**  **1h** | -0.268 | 0.265 | 0.021 | 0.311 | 0.134 | 0.276 | 0.439 | 0.192 | 0.176 | 0.368 | 0.207 | 0.402 | 0.323 | 0.342 | **-.883^**^** | -0.270 |
| **mg/dl** | 0.254 | 0.259 | 0.930 | 0.182 | 0.731 | 0.472 | 0.204 | 0.620 | 0.651 | 0.330 | 0.565 | 0.284 | 0.435 | 0.452 | **0.008** | 0.558 |
| **LDH**  **6h** | -0.379 | 0.261 | -0.061 | 0.367 | 0.188 | 0.395 | 0.206 | 0.265 | 0.069 | 0.022 | 0.153 | 0.385 | -0.203 | 0.351 | -0.116 | -0.056 |
| **mg/dl** | 0.110 | 0.280 | 0.803 | 0.123 | 0.455 | 0.105 | 0.427 | 0.287 | 0.785 | 0.932 | 0.545 | 0.114 | 0.527 | 0.263 | 0.720 | 0.862 |
| **LDH**  **12h** | 0.042 | 0.035 | 0.133 | 0.545 | -0.176 | 0.224 | 0.318 | 0.285 | 0.006 | 0.309 | 0.227 | -0.042 | 0.357 | -0.095 | 0.167 | 0.429 |
| **mg/dl** | 0.897 | 0.914 | 0.681 | 0.067 | 0.627 | 0.533 | 0.340 | 0.425 | 0.987 | 0.385 | 0.502 | 0.907 | 0.385 | 0.823 | 0.693 | 0.289 |
| **LDH**  **end** | -0.220 | 0.059 | -0.023 | 0.356 | -0.248 | 0.273 | 0.327 | 0.297 | 0.018 | 0.285 | 0.255 | -0.055 | 0.310 | -0.048 | 0.024 | 0.476 |
| **mg/dl** | 0.381 | 0.817 | 0.929 | 0.147 | 0.489 | 0.446 | 0.326 | 0.405 | 0.960 | 0.425 | 0.450 | 0.881 | 0.456 | 0.911 | 0.955 | 0.233 |
| **ph**  **1h** | 0.350 | -0.287 | 0.385 | -0.350 | -0.245 | 0.039 | 0.164 | 0.081 | 0.005 | -0.034 | 0.047 | -0.211 | -0.309 | 0.327 | 0.082 | 0.045 |
| **mg/dl** | 0.265 | 0.366 | 0.217 | 0.265 | 0.343 | 0.881 | 0.558 | 0.758 | 0.985 | 0.896 | 0.863 | 0.417 | 0.355 | 0.326 | 0.811 | 0.894 |
| **ph**  **6h** | 0.290 | 0.001 | -0.116 | -0.447 | -0.387 | 0.096 | 0.211 | 0.157 | 0.152 | -0.181 | -0.024 | -0.208 | -0.236 | 0.345 | -0.036 | -0.064 |
| **mg/dl** | 0.243 | 0.997 | 0.648 | 0.063 | 0.125 | 0.715 | 0.451 | 0.548 | 0.560 | 0.486 | 0.931 | 0.422 | 0.484 | 0.298 | 0.915 | 0.853 |
| **ph**  **12h** | 0.282 | -0.209 | -0.473 | **-.845^**^** | -0.248 | 0.224 | 0.300 | 0.345 | -0.079 | 0.236 | 0.145 | 0.236 | 0.452 | -0.214 | -0.095 | 0.167 |
| **mg/dl** | 0.401 | 0.537 | 0.142 | **0.001** | 0.489 | 0.533 | 0.370 | 0.328 | 0.829 | 0.511 | 0.670 | 0.511 | 0.260 | 0.610 | 0.823 | 0.693 |
| **ph**  **end** | 0.204 | 0.079 | 0.089 | 0.436 | -0.414 | 0.042 | 0.079 | 0.074 | 0.042 | -0.228 | 0.012 | -0.152 | -0.227 | 0.200 | 0.182 | -0.064 |
| **mg/dl** | 0.467 | 0.781 | 0.751 | 0.104 | 0.098 | 0.874 | 0.781 | 0.779 | 0.874 | 0.379 | 0.966 | 0.560 | 0.502 | 0.555 | 0.593 | 0.853 |
| **Lactate 1h** | -0.099 | 0.296 | -0.017 | 0.215 | 0.367 | -0.333 | -0.358 | -0.583 | 0.233 | -0.400 | -0.079 | 0.050 | 0.286 | -0.071 | 0.250 | 0.429 |
| **mg/dl** | 0.677 | 0.204 | 0.945 | 0.362 | 0.332 | 0.381 | 0.310 | 0.099 | 0.546 | 0.286 | 0.829 | 0.898 | 0.493 | 0.879 | 0.589 | 0.337 |
| **Lactate 6h** | -0.081 | 0.188 | -0.268 | 0.193 | 0.121 | 0.044 | 0.275 | 0.330 | 0.253 | -0.275 | -0.021 | -0.511 | 0.167 | -0.394 | 0.394 | 0.164 |
| **mg/dl** | 0.750 | 0.454 | 0.282 | 0.442 | 0.694 | 0.887 | 0.342 | 0.271 | 0.405 | 0.364 | 0.940 | 0.074 | 0.693 | 0.260 | 0.260 | 0.651 |
| **Lactate 12h** | -0.082 | 0.059 | 0.452 | 0.100 | 0.134 | 0.276 | 0.439 | 0.192 | 0.176 | 0.368 | 0.207 | 0.402 | 0.323 | 0.342 | **-.883^**^** | -0.270 |
| **mg/dl** | 0.810 | 0.862 | 0.163 | 0.769 | 0.731 | 0.472 | 0.204 | 0.620 | 0.651 | 0.330 | 0.565 | 0.284 | 0.435 | 0.452 | **0.008** | 0.558 |
| **Lactate end** | 0.174 | 0.303 | -0.108 | 0.121 | 0.188 | 0.395 | 0.206 | 0.265 | 0.069 | 0.022 | 0.153 | 0.385 | -0.203 | 0.351 | -0.116 | -0.056 |
| **mg/dl** | 0.463 | 0.195 | 0.651 | 0.611 | 0.455 | 0.105 | 0.427 | 0.287 | 0.785 | 0.932 | 0.545 | 0.114 | 0.527 | 0.263 | 0.720 | 0.862 |

*Spearman-rank correlation coefficient with according p-value. Statistically, significant correlations are marked in bold*

*ALT,* Alanine Aminotransferase*; AST, Aspartate Aminotransferase; LDH, lactate dehydrogenase; StO2, Tissue Oxygen Saturation; THI, Tissue Hemoglobin Index; NIR, Near-Infrared Perfusion Index; TWI, Tissue Water Index*

**References**

1. Cardini B, Oberhuber R, Fodor M, et al. Clinical Implementation of Prolonged Liver Preservation and Monitoring Through Normothermic Machine Perfusion in Liver Transplantation. *Transplantation*. 2020.

2. Sucher R, Wagner T, Köhler H, et al. Hyperspectral Imaging (HSI) of Human Kidney Allografts. *Ann Surg*. 2020.

3. Moulla Y, Buchloh DC, Köhler H, et al. Hyperspectral Imaging (HSI)-A New Tool to Estimate the Perfusion of Upper Abdominal Organs during Pancreatoduodenectomy. *Cancers (Basel)*. 2021; **13**.

4. Holmer A, Marotz J, Wahl P, Dau M, Kämmerer PW. Hyperspectral imaging in perfusion and wound diagnostics - methods and algorithms for the determination of tissue parameters. *Biomed Tech (Berl)*. 2018; **63**: 547-56.
